# Cardiorespiratory effects of recruitment maneuvers and positive end expiratory pressure in an experimental context of acute lung injury and pulmonary hypertension

**DOI:** 10.1186/s12890-015-0079-y

**Published:** 2015-07-31

**Authors:** Camille Doras, Morgan Le Guen, Ferenc Peták, Walid Habre

**Affiliations:** Anesthesiological Investigation, University Medical Centre, University of Geneva, Geneva, Switzerland; Department of Anesthesiology, Hospital Foch, University Versailles Saint-Quentin en Yvelines, Suresnes, France; Department of Medical Physics and Informatics, University of Szeged, Szeged, Hungary; Pediatric Anesthesia Unit, Geneva Children’s Hospital, Rue Willy Donzé 6, 1205 Geneva, Switzerland

## Abstract

**Background:**

Recruitment maneuvers (RM) and positive end expiratory pressure (PEEP) are the cornerstone of the open lung strategy during ventilation, particularly during acute lung injury (ALI). However, these interventions may impact the pulmonary circulation and induce hemodynamic and respiratory effects, which in turn may be critical in case of pulmonary hypertension (PHT). We aimed to establish how ALI and PHT influence the cardiorespiratory effects of RM and PEEP.

**Methods:**

Rabbits control or with monocrotaline-induced PHT were used. Forced oscillatory airway and tissue mechanics, effective lung volume (ELV), systemic and right ventricular hemodynamics and blood gas were assessed before and after RM, during baseline and following surfactant depletion by whole lung lavage.

**Results:**

RM was more efficient in improving respiratory elastance and ELV in the surfactant-depleted lungs when PHT was concomitantly present. Moreover, the adverse changes in respiratory mechanics and ELV following ALI were lessened in the animals suffering from PHT.

**Conclusions:**

During ventilation with open lung strategy, the role of PHT in conferring protection from the adverse respiratory consequences of ALI was evidenced. This finding advocates the safety of RM and PEEP in improving elastance and advancing lung reopening in the simultaneous presence of PHT and ALI.

## Background

Several pathophysiological mechanisms contribute to the development of atelectasis during mechanical ventilation with consecutive loss of lung volume and hypoxia [1]. The promoted ventilation strategy “open the lung and keep it open” [2, 3] is based on the application of recruitment maneuvers (RM) followed by the maintenance of a positive end-expiratory pressure (PEEP) [4]. Several techniques of RM are discussed in the literature but they all consist in achieving, repeatedly and for a specified period of time, an insufflation pressure corresponding to the total lung capacity [5–8].

In the presence of acute lung injury (ALI) pulmonary capillaries are damaged by increased permeability [9] and the alveoli are compressed by diffuse edema and inflammation. Under this condition, lung-protective ventilation strategy designed to open the lung is of paramount to maintain effective lung volume and oxygenation [10]. Despite the limited evidence for improvement in mortality in the presence of ALI [11], high PEEP and RM may have short term benefit to patients maintained on ventilatory support for the treatment of all spectrum of ALI [12–17]. However, recruiting the lung and applying high PEEP increase intra-thoracic pressure and may lead to alveolar overdistension, with consequent increase in physiological dead space [18–20]. Another limit is the compromised venous return, thereby counteracting the hemodynamic balance [21–23]. Hence, the right circulation is particularly exposed with a risk for alteration in pulmonary afterload, cardiac output, stroke volume and compromised gas exchange [13, 24, 25].

The altered pulmonary circulation in patients with pulmonary arterial hypertension (PHT) adds to the complexity of the optimization of ventilation strategy [26, 27]. While RM and PEEP compromise right heart function via increasing the intra-thoracic pressure, major hemodynamic and respiratory effects of open lung strategy could be anticipated during concomitant presence of ALI and PHT [28]. Although the individual pathophysiological consequences of ALI and PHT have been extensively investigated, the effects of their coexistence on the cardiorespiratory function during RM at different PEEP levels have not been fully explored.

Therefore, we aimed at characterizing the effects of RM and PEEP on the cardiorespiratory function during lung injury induced by surfactant depletion in an experimental model of PHT. The pressure transmission across the alveolo-capillary membrane is blunted in the presence of compromised lung compliance due to the limited expansion of the lung periphery, such as observed during ALI. Moreover, the over pressurized capillaries in the presence of PHT may further attenuate the pressure gradient across the alveolar capillary wall. Thus, it can be hypothesized that RM and PEEP will lead to less deleterious effects on pulmonary hemodynamics and cardiac function in the presence of ALI and PHT.

## Materials and methods

### Ethical approval

All experiments and procedures were conducted under the agreement of the Swiss animal welfare committee (Geneva Cantonal Veterinary Office registration number 1051/3890/2). 19 females New Zealand White rabbits of about 4 month-old (3.1 ± 0.1 kg) were used.

### Animal preparations

The rabbits were randomly assigned into two groups. Pulmonary hypertension was induced in the animals of the PHT group by a single intravenous dose of 60 mg/kg of monocrotaline (Sigma-Aldrich) prepared in acidized PBS with adjusted pH around 7.4 [29–33]. Animals of the control group (CTRL) received only the solvent (PBS) at an equivalent volume. 21 days later, the animals were enrolled into the final blinded procedure as followed.

Rabbits were sedated with an intramuscular administration of xylazine 2 % (5 mg/kg). After 15 minutes anesthesia was induced by intravenous injection of midazolam diluted to 0.2 % (3–6 mg/kg) via a catheter introduced into an ear vein. The animals were then tracheostomised under local anesthesia (Xylocaine 0.5 % 1 mL subcutaneous) and mechanically ventilated with pressure-regulated volume controlled (PRVC) mode, with a target tidal volume of 5 to 7 ml/kg, by using a neonatal ventilator (Servo-I, Maquet Critical Care, Solna Sweden). Respiratory rate was set around 30-40/min with an inspiration/expiration ratio of 1:2, in order to achieve an end-tidal carbon dioxide (etCO_2_) around 5 %. The initial inspired oxygen fraction (FiO_2_) was set to 40 %. The PEEP was set at 3 cmH_2_O during surgery. Anesthesia was maintained throughout the experiment by a continuous infusion of a mixture of midazolam (0.6-0.75 mg/kg/h), fentanyl (20–25 μg/kg/h) and atracurium besylate (1–1.25 mg/kg/h) conveyed in 0.9 % saline.

The left carotid artery and the right jugular vein were catheterized (20 gauge catheter) for blood sampling and arterial and central venous pressure monitoring. Body temperature was continuously controlled and maintained around 38-39 °C by applying a heating pad. Electrocardiogram, blood and tracheal pressures and right ventricular PV loops were continuously collected and recorded via PowerLab data acquisition hardware, and computerized with LabChart software (ADinstrument, Dunedin, New Zealand). Low frequency forced oscillatory respiratory mechanics, venous and arterial blood gas (VetScan i-STAT1 Handheld Analyzer with EG6+ cartridge, Abaxis, Union City, CA, USA) and effective lung volume were collected and registered at specified time points as described thereafter.

### Study Protocol

**Fig. 1:**
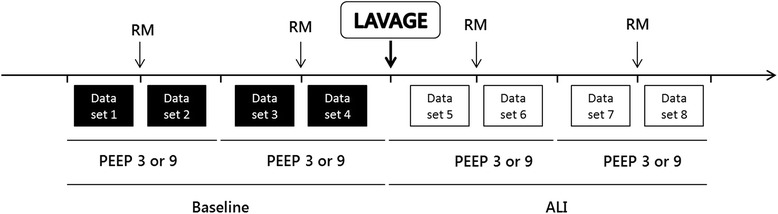
Experimental protocol

The experimental protocol (Fig. 1) consisted of collection of data sets before and after RM under baseline (BASAL) and acute lung injury (ALI) with maintenance of low (3 cmH_2_O) or high (9 cmH_2_O) PEEP levels. The two PEEP levels were applied in a randomized order. Each data set consisted in collecting the following sequence: hemodynamic parameters, respiratory impedance, effective lung volume and blood gas. After reaching steady-state conditions while rabbits were ventilated with a particular PEEP, a first set of data was collected and considered as baseline before RM. Standardized RM were then performed in pressure control mode, in achieving three hyperinflations (inspiratory pause) of 27 cmH_2_O above PEEP for five seconds, repeated every ten seconds [6, 34, 35]. 1 min after RM, a second set of data was collected as baseline after RM. The second PEEP level was next set, and the same experimental procedure was repeated. ALI was then generated by whole-lung lavage with 0.9 % saline solution heated at 38 ° C, with a volume of 20 ml/kg instilled five times at five minutes interval via the endotracheal tube by gentle mechanical push. Lung fluid was then withdrawn by gentle manual suctioning and its volume was measured. Following lavage, the animal was stabilized for 15 min during which FiO_2_ was increased to 60 % and respiratory rate raised if needed, to maintain adequate gas exchange with PaO_2_ > 10 kPa and PaCO_2_ of less than 6 kPa. Full sets of measurements were then repeated as under the basal condition, with a new randomized order in PEEP. At the end of the experiment and under continuous anesthesia, the animals were euthanized by an intravenous injection of pentobarbital (lethal dose 150 mg/kg). Then the heart-lung block was removed and stored in neutral buffered formalin for subsequent analyses.

### Right ventricle hemodynamics

An admittance catheter (Scisense 3.5 F Medium Rabbit Variable segment length Transonic, Ithaca, NY, USA) was placed into the right ventricle via the right jugular vein and connected to a dedicated signal conditioner system (Scisense ADVantage Pressure-Volume control unit, ADV500 System, Transonic, Ithaca, NY, USA), linked to PowerLab data acquisition unit. The magnitude and phase of the electrical admittance as well as the right ventricle pressure and volume were continuously monitored and analyzed on LabChart software. Right ventricle pressure and volume at the end of the diastole and at the end of the systole were extracted from the recordings over specified period of time as average cyclic peak values. Stroke volume was obtained by subtracting end systolic volume from end diastolic volume cardiac output was calculated as the product of stroke volume multiplied by heart rate. The parameters were normalized by the body weight when appropriate.

### Respiratory mechanical measurements

The forced oscillation technique using low frequencies was applied to measure the airway and respiratory tissue mechanical parameters as detailed previously [36]. Briefly, small-amplitude (1 cmH_2_O peak to peak) pressure forcing signal (0.5-21 Hz) generated by a loudspeaker-in-box system was driven to the trachea via a polyethylene tube (100 cm length, 0.375 cm ID) while the mechanical ventilation was paused at end-expiration. The loudspeaker chamber was pressurized to the level of PEEP in order to maintain pressure constant during the recordings. Lateral pressures were measured at the loudspeaker end (P_1_) and the tracheal end (P_2_) of the wave-tube with miniature pressure transducers (ICS 33NA00D, Milpitas, CA, USA). These pressure signals were low-pass filtered (corner frequency of 25 Hz) and digitized at a sampling rate of 128 Hz. The pressure transfer function (P_1_/P_2_) was calculated by fast Fourier transformation from the 8 s recordings and the input impedance of the respiratory system (Zrs) was computed from this pressure transfer function as the load impedance of the wave-tube [37]. Three to five Zrs spectra were ensemble-averaged under each experimental condition.

To separate airway and respiratory tissue mechanics from Zrs spectra a model containing frequency-independent airway resistance (Raw) and inertance (Iaw), in series with a constant-phase tissue model [38] including damping (G) and elastance (H) was fitted to Zrs by means of a global optimization procedure. As previously established, Raw reflects mainly the flow resistance of the airways, Iaw is related to the cyclic acceleration and deceleration of the intra-thoracic gas, G describes the energy loss within the respiratory tissues (resistance) whereas H characterizes the energy storage capacity of the respiratory tissues (elastance). The reported Raw and Iaw values were corrected by removing tracheal setup contribution.

### Measurement of effective lung volume by Differential Fick Method

ELV, defined as the lung volume taking part into the gas exchange, was assessed as described earlier [39, 40]. Briefly, periods of five consecutive alterations in inspiratory/expiratory ratio (1:2–1.5:1) were applied by the ventilator. This specific breathing pattern varies etCO_2_ of approximately 0.5–1.0 kPa, which allows estimation of ELV using the differential Fick equation. Rabbit flow and expired CO_2_ were measured by the ordinary Y-piece flow sensor and the main stream CO_2_-transducer in Servo-i. Flow and CO_2_ data from Servo-i were exported to a laptop with a specially designed software application written in Matlab™ (Mathworks, Natick, MA).

### Histology

Lungs kept into formalin were processed by conventional histology with hematoxylin and eosin staining of 10 μm paraffin sections.

### Statistical Analyses

Group means with standard error values are reported. Normal distribution was verified with the Shapiro-Wilk test. Logarithmic transformation was applied to normalize data where appropriate. The significance of change in values before and after RM was tested by using two-way repeated measures ANOVA with Sidak pairwise multiple comparison procedure. Three-way ANOVA tests with Holm-Sidak pairwise multiple comparisons were performed on absolute values to analyse the effect of PHT and PEEP within basal or ALI conditions as well as to analyze the effect of ALI within both PEEP. The statistical tests were performed with SigmaPlot (Version 12.5, Systat Software, Inc.) or Prism (version 6, GraphPad Software Inc.).

## Results

Over the initial pool of 19 rabbits, animals were excluded for some parameters for technical or medical reasons. Seven rabbits were excluded for the right ventricle PV measurement due to defects of the catheter, and three animals were excluded from all data because of systemic failures during anesthesia. Consequently, 12 rabbits were included in the analyses of the ventricular PV data (6 in each group), and 16 animals were included in all other examinations (8 in each groups).

### Hemodynamic changes

**Fig. 2:**
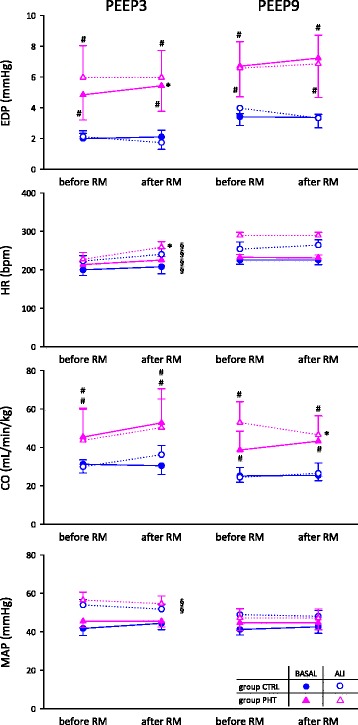
Right ventricle end diastolic pressure (EDP), heart rate (HR), weighted cardiac output (CO) and mean arterial pressure (MAP), before and after RM, during baseline (black symbols) or ALI (open symbols), in control (circle) and PHT (triangle) animals, at 2 levels of PEEP. Statistical relevance of * RM, # PHT, § PEEP

Figure 2 demonstrates the hemodynamic parameters obtained in CTRL and PHT groups in healthy lungs and following surfactant depletion, during the maintenance of two PEEP levels, before and after RM. The significant effects of PHT, PEEP and RM are reported on graphs. In all experimental conditions, monocrotaline treatment induced PHT consistently with more than twofold increase in the end diastolic pressure (EDP) (# p = 0.003) and a significant increase in cardiac output (CO) (# p = 0.04), compared to control, while it generated no statistically detectable change in the other hemodynamic parameters. ALI significantly increased heart rate (HR) at both PEEP levels (p < 0.01) and mean arterial pressure (MAP) during PEEP3 (p < 0.001). High PEEP increased HR in all animals and decreased MAP in ALI animals only (§ p < 0.05). Finally RM elevated the EDP in PHT group while the lower PEEP was maintained (22.6 ± 12.2 %, * p = 0.005). RM also induced significant changes in some hemodynamic parameters in the concomitant presence of ALI and PHT: HR under PEEP3 (16.0 ± 6.1 %, * p = 0.01) and CO under PEEP9 (-11.4 ± 4.8 %, * p = 0.02).

### Changes in respiratory function

**Fig. 3:**
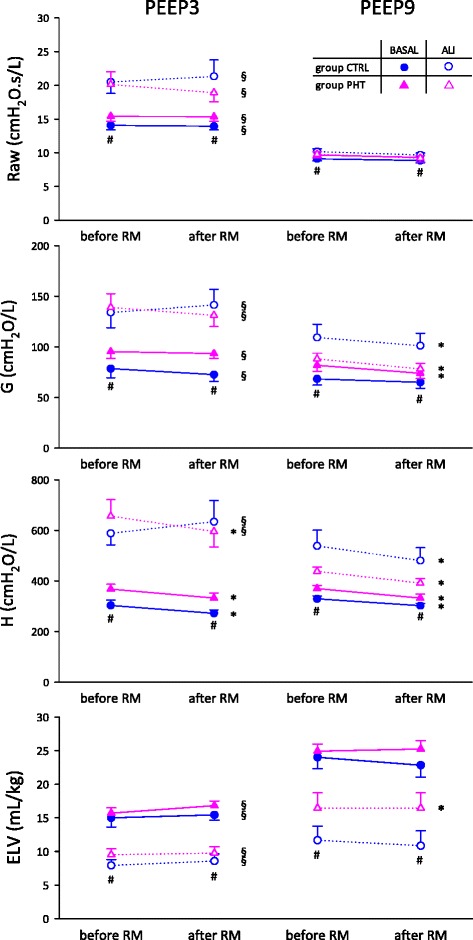
Airway resistance (Raw), tissue damping (G), tissue elastance (H) and effective lung volume (ELV), before and after RM, during baseline (black symbols) or ALI (open symbols), in control (circle) and PHT (triangle) animals, at 2 levels of PEEP. Statistical relevance of * RM, # PHT, § PEEP

**Fig. 4:**
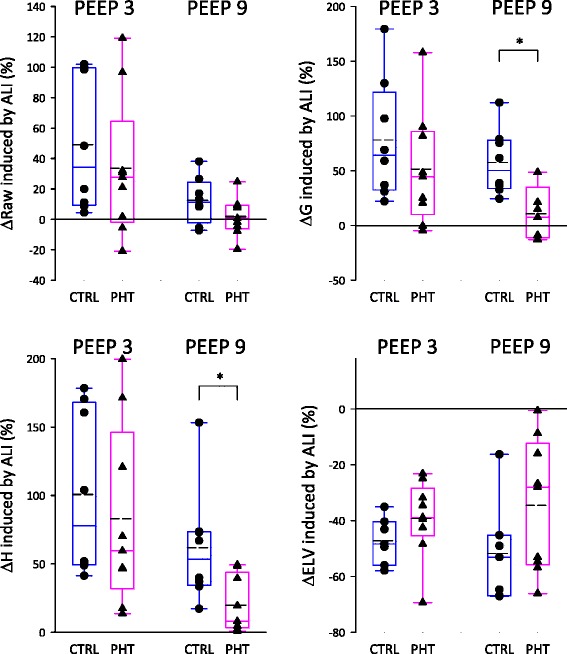
Comparison of the relative change induced by ALI in Raw, G, H and ELV between control and PHT rabbits, at 2 levels of PEEP

Figure 3 depicts respiratory mechanical changes in protocol groups under the different experimental conditions. During baseline, Raw, G and H were significantly higher in PHT group than in controls (# p = 0.03, p = 0.001, p < 0.001), whereas ELV was not different between groups. However ELV was significantly higher in the presence of PHT following whole lung lavage (# p = 0.005). Furthermore, induction of ALI significantly compromised all respiratory mechanics as well as ELV, at both PEEP levels (p < 0.001 for all), except for Raw at the higher PEEP. To further characterize how the presence of PHT influenced the lung consequences of ALI, the relative changes in the forced oscillatory and ELV parameters following lung lavage were assessed (Fig. 4). ALI-induced changes in Raw were not different between groups at any PEEP level. However, the relative changes in G and H induced by ALI were significantly reduced by the presence of PHT at PEEP9 (* p = 0.005 and * p = 0.002). Regarding ELV, ALI tended to have less impact in the PHT group, with a difference close to statistical significance (p = 0.06).

Figure 3 also shows that the application of a high PEEP significantly improved all forced oscillatory parameters as well as ELV (§ < 0.01) in both groups during baseline and ALI, except for H during baseline. Finally RM demonstrated efficiency in decreasing H under both PEEP levels and each experimental condition (* p < 0.05), except for the control group following ALI. During RM, G also exhibited a significant decrease at PEEP9 in almost every conditions (* p < 0.03). At PEEP9, the application of RM in group PHT elevated ELV (7.2 ± 1.6 %, * p = 0.02).

### Gas exchange parameters

**Fig. 5:**
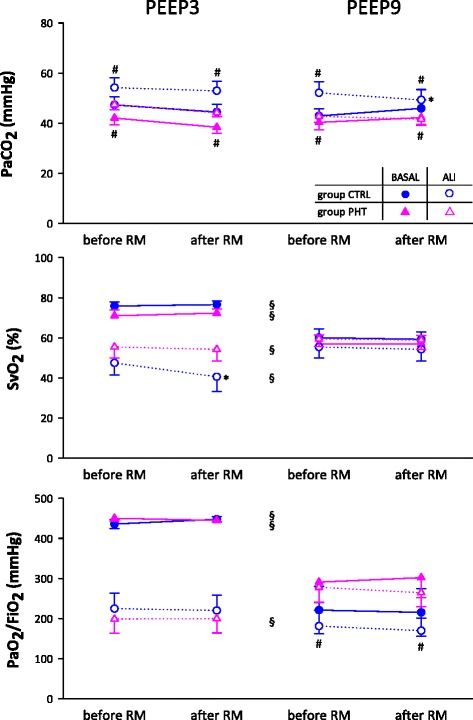
Carbon dioxide arterial partial pressure (PaCO_2_), oxygen venous saturation (SvO_2_) and oxygen arterial partial pressure / inspired fraction ratio (PaO_2_/FiO_2_), before and after RM, during baseline (black symbols) or ALI (open symbols), in control (circle) and PHT (triangle) animals, at 2 levels of PEEP. Statistical relevance of * RM, # PHT, § PEEP

The presence of PHT significantly decreased PaCO_2_ during baseline and ALI and under both PEEP levels (# p < 0.04), while it increased the PaO_2_/FiO_2_ ratio in the only case of animals experiencing ALI under PEEP9 (# p = 0.01) (Fig. 5). When PEEP3 was maintained, surfactant depletion compromised PaCO_2_, SvO_2_ and PaO_2_/FiO_2_ (p < 0.01), whereas these adverse changes were not detectable at PEEP9. Increasing PEEP had a pejorative effect on SvO_2_ and PaO_2_/FiO_2_ during baseline (§ p < 0.001) but during ALI this ameliorated SvO_2_ (§ p = 0.003). Moreover, there was an improvement in PaO_2_/FiO_2_ in the concomitant presence of ALI and PHT (§ p = 0.04) but not in the sole presence of lung injury. Finally during the ALI sequence in group CTRL, RM decreased PaCO_2_ at the higher PEEP (−5.3 ± 1.0 %, * p = 0.003) and SvO_2_ at the lower PEEP (−18.3 ± 5.2, * p = 0.002).

## Discussion

During mechanical ventilation, applying an open lung strategy by performing recruitment maneuvers under the maintenance of two PEEP levels demonstrated beneficial respiratory effects in lungs with physiological surfactant function, with no evidence for major hemodynamic impairment, regardless of the presence of PHT. In the injured lungs however, lung recruitments proved to be more efficient in improving respiratory elastance and lung volume in the concomitant presence of PHT. Moreover, PHT blunted the adverse respiratory mechanical and lung volume consequences of surfactant depletion when sufficient PEEP was maintained to target the open lung strategy.

In agreement with previous findings using similar animal models [31, 41–43], monocrotaline treatment in the present study led to the development of plexogenic PHT manifested in elevated right ventricle EDP and pulmonary vascular remodeling. Moreover, the deleterious consequences of monocrotaline administration on the airway and respiratory tissue mechanical parameters are in accordance with earlier results [44, 45].

As concerns the applied model of ALI by surfactant depletion, ventilation with the lower PEEP led to consequent blood gas impairment, compromised airway and respiratory tissue mechanics and diminished ELV [39, 46, 47]. While this model mimics the loss of surface forces without involving other important pathologies related to ALI, these adverse alterations are in accordance with the Berlin definition for a mild ARDS [48]. In line with previous observation, increasing PEEP in the injured lungs prohibited these adverse effects [11, 49–52]. Since hemodynamic function was preserved after lavage, deleterious blood gas alterations are likely to be linked to ventilation defects provoked by surfactant depletion.

The most remarkable finding of the present study is the inhibition of the adverse respiratory mechanical and lung volume consequences of surfactant depletion in the animals with PHT at the higher PEEP level (Fig. 4) and the improved effectiveness of RM in reversing the adverse consequences of surfactant depletion (Figs. 3 and 5). Regarding the pathophysiological mechanisms responsible for these findings the coexistence of various processes can be anticipated.

First of all, PHT leads to adverse alterations in lung viscoelasticity for structural and hemodynamic reasons. Structural elements are related to the thickening of the pulmonary capillary walls, which has been demonstrated to occur in human PHT as in monocrotaline animal models [32]. We also found pulmonary vessels with histological evidences for hypertrophy and hyperplasia in external layers (media and adventice) with further cross-sectional area restriction in the hypertensive lungs (data not shown). Then hemodynamic reasons rely on the fact that PHT increases H via cardiopulmonary interactions. Indeed, higher pulmonary arterial pressure increases retraction forces, therefore exerting a tethering effect on the alveoli that provides support to the lung architecture [53, 54]. Therefore, we suggest that in monocrotaline PHT rabbits, this greater mechanical tensile strength of the alveolar capillary network, along with enhanced elastic recoil forces, prohibited alveolar closures and facilitated recruitment.

A further involvement of the cardiopulmonary interactions in the protection of PHT against the adverse consequences of ALI as well as the stress failure of RM during ALI, can be anticipated in view of adverse regional changes following surfactant depletion. By applying functional imaging technique, we recently provided experimental evidence for a heterogeneous alveolar derecruitment after surfactant depletion [46]. These circumstances explain the adverse respiratory mechanical changes and the decreases in ELV obtained in the present study. The loss of ventilation ultimately leads to compromised perfusion of these lung regions due to hypoxic vasoconstriction [55]. Since PHT obviously maintains high filling pressure in the pulmonary capillaries, the lung perfusion may be better preserved in the under-aerated regions and thus, the adverse consequences of ALI are blunted. This phenomenon can be appreciated from the strong tendency for an improvement in lung oxygenation after increasing PEEP in the concomitant presence of ALI and PHT. The beneficial effect of alveolar recruitment overwhelmed the detrimental effects of alveolar overdistension at high PEEP (Fig. 5). This concept is also in line with earlier findings demonstrating that an elevated carbon monoxide diffusion capacity reflecting increased pulmonary capillary blood volume is associated with an improved response to high PEEP [56] and a better survival outcome in ARDS patients [57].

A similar concept has been revealed previously by Kornecki *et al.* demonstrating that rats with PHT were less prone to ventilation induced lung injury than normotensive rats [58]. Along with our findings worsening of oxygenation, respiratory compliance and edema development were less pronounced in the presence of pulmonary vascular remodeling. There are some similarities between ALI and VILI in the sense that mechanical strains play a key role in their pathogenesis in contributing to the disruption in the alveolar capillary coupling [26, 58–60]. Thus, the present study provides further evidence on the role of PHT in protecting the lungs against mechanical strains, regardless of the source of injury.

## Conclusions

Application of RM and the maintenance of high PEEP are widely accepted as key factors in the concept of open lung strategy during mechanical ventilation. The present study addressed the respiratory and hemodynamic consequences of this ventilation strategy in an animal model with concomitant presence of ALI and PHT. As a novel finding, we evidenced the role of PHT in conferring protection from the adverse respiratory consequences of ALI with more favorable profile of RM in improving elastance and advancing lung reopening. With the precaution of extrapolating experimental findings to clinical settings, the results of the present study may imply that adaptation of open lung strategy can be safely considered even in the presence of PHT.

## Abbreviations

ALI: Acute Lung Injury
ARDS: Acute respiratory distress syndrome
CO: Cardiac output
EDP: End-diastolic pressure
ELV: Effective lung volume
etCO_2_: End-tidal carbon dioxide
FiO_2_: Inspired oxygen Fraction
G: Tissue damping
H: Tissue elastance
Iaw: Airway inertance
HR: Heart rate
MAP: Mean arterial pressure
PaCO_2_: CO_2_ Concentration in the arterial blood
PaO_2_: O_2_ Concentration in the arterial blood
PEEP: Positive end expiratory Pressure
PHT: Pulmonary arterial hypertension
PRVC: Pressure-regulated volume controlled
Raw: Airway resistance
RM: Recruitment maneuver
Zrs: Input impedance of the respiratory system
